# Septate gallbladder in the laparoscopic era

**DOI:** 10.4103/0972-9941.40994

**Published:** 2008

**Authors:** Nitin R Patel, Vismit P Joshipura, Sanjiv P Haribhakti, Harshad N Soni

**Affiliations:** Department of Gastrointestinal and Laparoscopic Surgery, Sterling Hospital, Sterling Hospital Road, Memnagar, Ahmedabad, Gujarat, India

**Keywords:** Gallbladder anomalies, laparoscopic treatment, septate gallbladder

## Abstract

The anatomy facing a surgeon during cholecystectomy is challenging as it involves complex relationship between the gallbladder, hepatic artery and extra-hepatic billiary tree. We report a case of septate gall bladder which was successfully treated with laparoscopic cholecystectomy. In this paper, we also discuss the embryology and characteristics of this rare anomaly. Lack of awareness, non-specific symptoms, signs and inadequacy of imaging methods are possible reasons for the reported problem of overlooking of this entity. Complete identification and removal of gallbladder is mandatory, as a remnant may result in recurrence of symptoms or stones.

## INTRODUCTION

Congenital anomalies of the gallbladder are rare and wide range of malformations pertaining to all aspects have been described.[1] Septate gallbladder has not been well documented because it is usually asymptomatic or discovered accidentally during the evaluation of abdominal pain.[1] Rarely due to septation there may be stone formation, which might lead to recurrent abdominal pain.[2] We report a case of septate gallbladder that underwent successful laparoscopic cholecystectomy (LC). This case illustrates the need for complete removal of gallbladder during surgery. Precise intraoperative recognition of vascular and billiary anatomy is necessary to avoid common bile duct (CBD) injuries and gallbladder remnants.

## CASE REPORT

A 55-year-old male patient was admitted to our hospital with a right upper quadrant pain recurring for the past 10 years. There was a history of postprandial fullness, belching and sour eructation's. There was no history of jaundice in past. His blood values were within normal range. Ultrasound (US) of upper abdomen showed the gallbladder with a Phrygian cap filled with multiple calculi. LC was planned.

**Figure 1:**
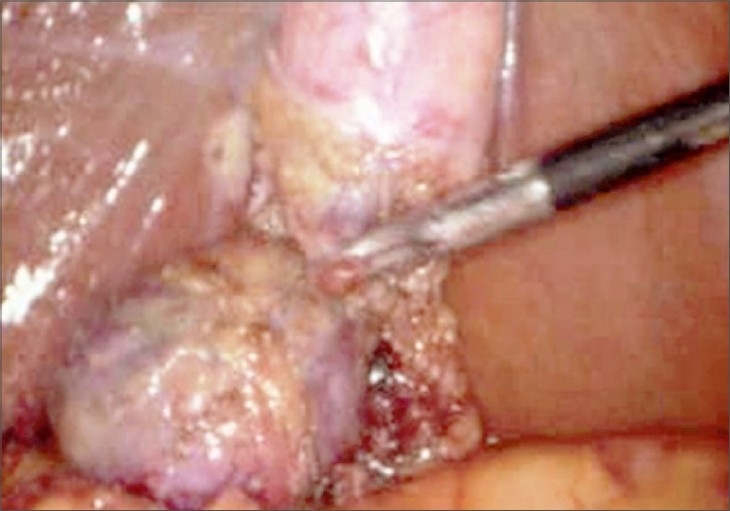
Septate gallbladder seen at laparoscopy

**Figure 2:**
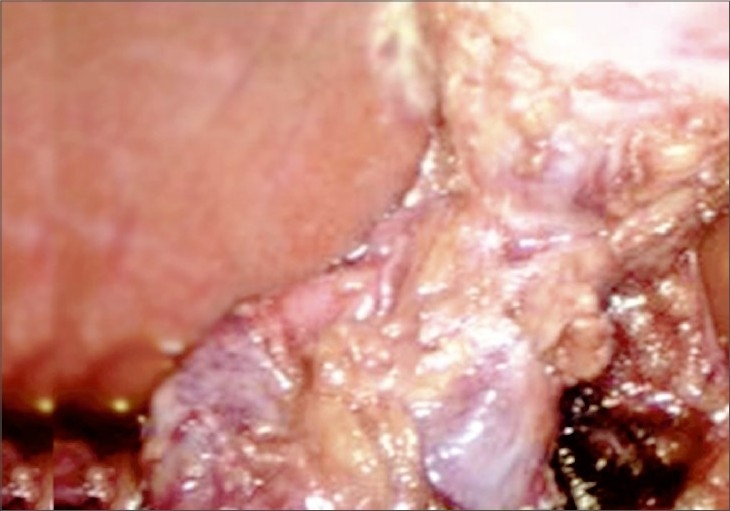
Completed dissection

At laparoscopy, flimsy adhesions were identified at the fundus of the gallbladder. After retracting the fundus towards the ipsilateral shoulder dense adhesions were present at the body of the gallbladder. After completing adhesinolysis with blunt and sharp dissection we found a septated structure [Figure 1]. Calot's triangle was dissected. Cystic artery and Cystic duct were clipped and divided. LC was successfully completed without any complication [Figure 2]. No drain was used in the patient. The duration of the operation was 40 min. The postoperative period was uneventful and the patient was discharged on the second postoperative day. On follow-up appointments, patient was in good condition.

**Figure 3:**
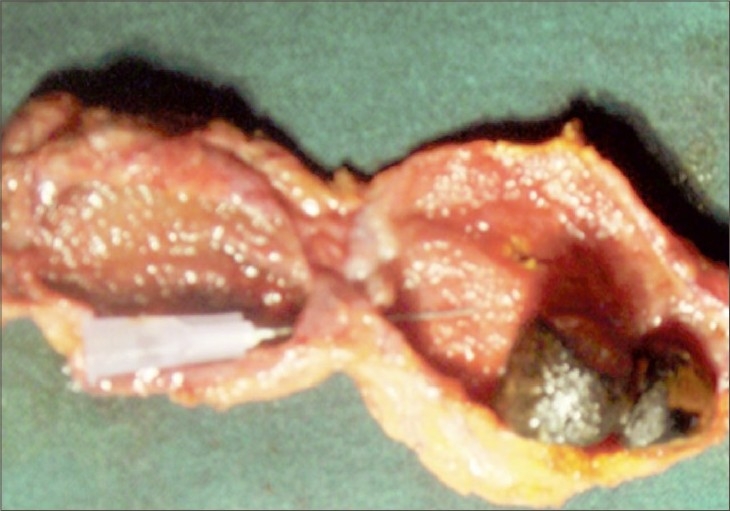
Specimen of septate gallbladder

Gross examination of the specimen [Figure 3] showed a horizontal septum with a pinhole communication. The cephalic moiety showed two stones and distal moiety was thin-walled and completely empty. There was evidence of acute inflammatory changes over entire wall.

## DISCUSSION

Anomalies of the gallbladder have been classified into malformation of shape, number, site, size and heteropias. These anomalies may be asymptomatic or may cause wide range of complications.

Septate gallbladder is characterized by the presence of a septum that divides the gallbladder in two chambers. When the septum dividing the gallbladder lies longitudinally it is called bilobed gallbladder and when there is a transverse septum separating the fundus from the rest of the gallbladder it is called an hour-glass gallbladder.[2] Septate gallbladders most likely result from incomplete resolution of the solid stage of gallbladder development that is present before the third month of fetal development. These gallbladder septae are most commonly single, but multi septate gallbladder, as well as post inflammatory adhesions and compartmentalization of the gallbladder have also been described.[3]

In our patient the septa were most probably congenital in origin with superadded inflammation which gave rise to classic septate shaped gallbladder. Secondly bile stasis and infection in the fundic part would have led to the development of the gallstones in this patient.

According the Boyden classification,[4] these congenital anomalies of gallbladder are classified according to the ductal formation as bilobed (or bifid), “V-shaped” and “H-shaped” (or ductular) gallbladder types. Classification of different types of double gallbladders is shown in Table 1.[5] The gallbladder in our case was compatible with the V-shaped one. To date US oral cholecystography oral cholecysto-computed tomography, scintigraphy, magnetic resonance cholangiography, percutaneous transhepatic cholangiography and endoscopic retrograde cholangiopancreatography have been used preoperatively to diagnose these anomalies.[6]

**Table 1:** Classification of different types of double gallbladder[5]

One cystic duct entering the common bile duct: Gall Bladder Septum (no evidence of duplication on the surface) Division of the gallbladder into two lobes joining at the neck to form a normal cystic duct (V duplication) Complete separation of the gallbladders, each with its own cystic ducts to join to form a common cystic (Y duplication) Two cystic ducts separately entering the bile ducts: Two cystic ducts entering the common bile ducts (H - duplication) One cystic duct entering the common bile duct, the other entering the right or left hepatic duct

Consequently, identification and definition of biliary anatomy is mandatory to prevent biliary system injury. Injury to the ductal system usually occurs during dissection of Calot's triangle. All connective tissue and fat must be removed completely to clearly expose the junction of the cystic duct with the gallbladder. We also performed meticulous dissection, keeping in mind principles of LC to prevent inadvertent injury. A careful appraisal of reported literature clearly emphasizes the need for removal of complete gallbladder to prevent surgical complications and repeated explorations. The author has treated two cases of stone in remnant of the gallbladder, after laparoscopic cholecystectomy.

In conclusion, this case illustrates the need for complete removal of gallbladder, as a remnant could be a good seat for development of stone in future and subsequent recurrence of symptoms.
